# Density-based clustering in haplotype analysis for association mapping

**DOI:** 10.1186/1753-6561-1-s1-s27

**Published:** 2007-12-18

**Authors:** Robert P Igo, Douglas Londono, Katherine Miller, Antonio R Parrado, Shannon RE Quade, Moumita Sinha, Sulgi Kim, Sungho Won, Jing Li, Katrina AB Goddard

**Affiliations:** 1 Department of Epidemiology and Biostatistics, Case Western Reserve University, Wolstein Research Building, 2103 Cornell Road, Room 1300-C, Cleveland, Ohio 44106, USA; 2 Department of Epidemiology, Johns Hopkins School of Public Health, 615 North Wolfe Street, Baltimore, Maryland 21205, USA; 3 Boehringer Ingelheim Pharmaceuticals, Inc., 900 Ridgebury Road, Ridgefield, Connecticut 06877, USA; 4 Department of Electrical Engineering and Computer Science, Case Western Reserve University, 10900 Euclid Avenue, Cleveland, Ohio 44106, USA; 5 Center for Health Research, Kaiser Permanente Northwest, 3800 N. Interstate Avenue, Portland, OR 97227, USA

## Abstract

Clustering of related haplotypes in haplotype-based association mapping has the potential to improve power by reducing the degrees of freedom without sacrificing important information about the underlying genetic structure. We have modified a generalized linear model approach for association analysis by incorporating a density-based clustering algorithm to reduce the number of coefficients in the model. Using the GAW 15 Problem 3 simulated data, we show that our novel method can substantially enhance power to detect association with the binary rheumatoid arthritis (RA) phenotype at the HLA-DRB1 locus on chromosome 6. In contrast, clustering did not appreciably improve performance at locus D, perhaps a consequence of a rare susceptibility allele and of the overwhelming effect of HLA-DRB1/locus C, 5 cM distal. Optimization of parameters governing the clustering algorithm identified a set of parameters that delivered nearly ideal performance in a variety of situations. The cluster-based score test was valid over a wide range of haplotype diversity, and was robust to severe departures from Hardy-Weinberg equilibrium encountered near HLA-DRB1 in RA case-control samples.

## Background

Haplotypes generally contain more information than individual single-nucleotide polymorphisms (SNPs) about the underlying genetic architecture, and therefore offer greater power to detect association between markers and traits. However, the power of haplotype-based methods for association mapping, like that of other approaches, is diminished in studies of complex traits by the presence of both allelic heterogeneity (i.e., mutations arising more than once in the same gene) and locus heterogeneity. One approach to ameliorate the effect of allelic heterogeneity is to cluster similar haplotypes, under the assumption that these may have diverged more recently in a population's history than the occurrence of a disease-causing mutation.

We combined the density-based clustering algorithm of Li and Jiang [1] with the general linear model (GLM) approach of Schaid et al. [2,3] for association mapping. Based on real pedigrees and SNPs, the simulated Genetic Analysis Workshop (GAW) 15 Problem 3 data sets provide an outstanding opportunity to compare the performance of our novel cluster-based method with the original, haplotype-based approach. The region near the HLA-DRB1 gene, in addition, presents an unusual context for rheumatoid arthritis (RA), on account of the very strong effect of certain HLA-DRB1 alleles on the phenotype [4], potentially inducing deviation from Hardy-Weinberg equilibrium (dHWE) in nearby SNPs in case-control samples. The GLM used in both methods relies on the assumption of HWE in calculating posterior probabilities of haplotype pairs from unphased SNP genotypes. The original approach of Schaid et al. appears to be robust to dHWE in simulated case-control data generated under a simple genetic model [5]. However, the sensitivity of our novel approach to dHWE remains to be tested.

In this report, we compare the performance of the haplotype- and cluster-based methods in detecting association with RA, and assess the type I error of both methods in the presence and absence of dHWE.

## Methods

### General methods

All analyses were carried out with knowledge of the true location of susceptibility loci.

Marker names from the chromosome 6 dense SNP scan are abbreviated here such that "denseSNP6_*N*" will be denoted as "SNP *N*". We tested the markers flanking the HLA-DRB1 locus (DRB1, coincident with SNP 3437, 49.5 cM) and locus D (between SNPs 3916 and 3917, 54.6 cM) for redundancy using BEST [6], and removed one redundant marker, SNP 3434, from the region near DRB1. We explored patterns of linkage disequilibrium (LD) and assessed the significance of nonzero LD by the likelihood ratio test in HaploView version 3.32 [7].

Testing for dHWE was carried out using the exact test in HaploView, and, for confirmation, the exact and χ^2 ^tests as implemented in the R genetics library package, version 1.2.0. Analyses were performed on sets of 1500 cases – one affected sib chosen at random from each affected-sib pair (ASP) family – and 2000 unrelated controls. All cases and controls in each set were from the same replicate.

We used the ASSOC program in S.A.G.E. [8] for case-control tests of association. The transmission disequilibrium test (TDT) was performed on trios of parents and affected child as implemented in the S.A.G.E. program TDTEX. Trios were selected with one random offspring from all 1500 ASP families in each replicate. In addition, the generalized family-based approach implemented in FBAT version 1.7.2 [9,10] was carried out in parallel on sets of 1500 complete ASP families, with a null hypothesis of no linkage or association.

### Association mapping by linear regression with clustering of haplotypes

We have extended the regression-based approach for association testing of Schaid et al. [3] to incorporate haplotype groups via a density-based clustering algorithm [1]. The primary goal was to reduce the dimensionality of the regression by clumping together haplotypes that are likely to have diverged recently, whether through mutation or recombination. Posterior haplotype probabilities from unphased data are obtained from the Decipher program in S.A.G.E. [8] and are imported into a modified version of the HapMiner program [1] as haplotype weights for clustering. Each pair of haplotypes is assigned a similarity score, a generalization of several scores previously described [11], which is converted to a distance metric on the interval [0,1] [1]. Clusters are formed in regions of high density (haplotype weight). A haplotype is designated a "core" haplotype if enough density, determined by the density threshold *MinPts*, is located within a given distance ε from it. Haplotypes within this ε neighborhood are clustered together. We modified HapMiner such that very common haplotypes, defined by the parameter *p*_min_, are never clustered together. This prevents improper grouping of ancient haplotypes. For the analyses presented here, we selected a value for *p*_min _of 1/(2*k*), where *k *is the number of haplotypes present with a frequency large enough to include in the GLM (see below).

Cluster assignments for all possible haplotypes are imported into the haplo.score function in HaploStats [2,3]. This method first estimates haplotype frequencies by the expectation maximization algorithm, and uses the frequencies to calculate posterior probabilities of haplotype pairs for each individual, assuming HWE. The posterior probabilities, in turn, are incorporated into a score test for association based on the likelihood function of a particular GLM – for case-control data, a logistic model – in which each haplotype is assigned a model coefficient [3]. A global score test for association is asymptotically distributed, under the null model of no association (all coefficients equal to 0), as a χ^2 ^random variable with degrees of freedom equal to the rank of the variance matrix for the score statistic. In our cluster-based approach, parameter estimates are obtained for clusters, rather than for haplotypes. We calculated the variance of the score statistic as per the generalized score test of Boos [12] as implemented by Tzeng et al. [13] because we found that variance calculation in Schaid et al. [3] based on the Louis information [14] inflated the type I error of the test when covariates were included in the analysis (data not shown). To prevent numerical instability and loss of power resulting from estimation of rare haplotypes [3], only haplotypes or clusters with frequencies above 0.002 were included.

### Power and type I error analyses on simulated datasets

We carried out studies of power and type I error of association mapping methods on subsets of all 100 replicates. Cases were randomly selected from the offspring of ASP families, such that no sample contained both sibs from any family; controls were chosen at random from the unrelated controls. All individuals within a sample were taken from the same replicate. We adjusted the sample size for each analysis to yield moderate (40–60%) power from the haplotype-only test. Where necessary, we included sex and the number of DRB1*04 alleles in the model as covariates, to reduce the signal strength to a level useful for power comparisons; this adjustment was not part of the analysis. Type I error in association-positive regions was assessed by randomizing case/control status (and covariates, if any) relative to genotype data by permutation.

A second null-model analysis was performed at locations far removed from the HLA region and locus D. The mean haplotype diversity, measured as the number of unique haplotypes present in the phased data, for all sets of six and eight consecutive markers was estimated over replicates 1–50 in two large regions on chromosome 6q comprising SNPs 11001–12000 and 15001–16000. Four levels of diversity were defined: low (10th percentile of all marker sets), medium (50th), high (90th) and very high (99th). Samples were extracted at selected locations at each diversity level, and score tests for association were carried out as above (without permutation).

## Results

### Deviation from HWE

**Figure 1:**
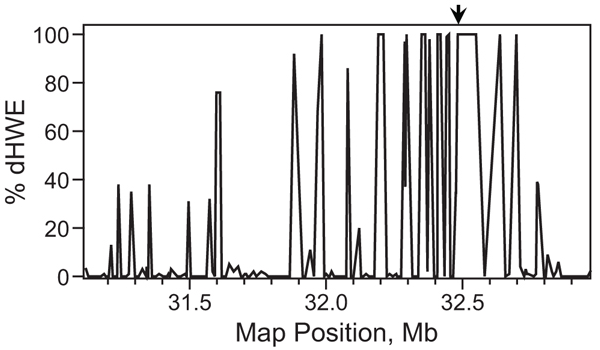
**Deviation from HWE in the neighborhood ofHLA-DRB1**. The χ^2 ^test for HWE was applied to samples from 100 replicates; the percentage of replicates in which *p *< 0.001 is shown for 170 consecutive markers near HLA-DRB1 (*arrow*).

We observed extensive dHWE in the neighborhood of HLA-DRB1 (Fig. 1). Forty-three SNPs within a region of 170 markers spanning from 31.1 Mb to 33.0 Mb on chromosome 6 showed significant dHWE, with *p *< 0.001 in at least five of the 100 replicates. Six consecutive markers flanking DRB1 gave *p*-values below 0.001 in every replicate and median *p*-values of less than 10^-6 ^from HaploView over all replicates. The three methods for testing HWE produced nearly identical results (data not shown). As expected, there was marked loss of heterozygosity in these markers. Because the score test for association assumes HWE, we took care to assess type I error for the two methods within this region (see below).

### Single-marker association

**Figure 2:**
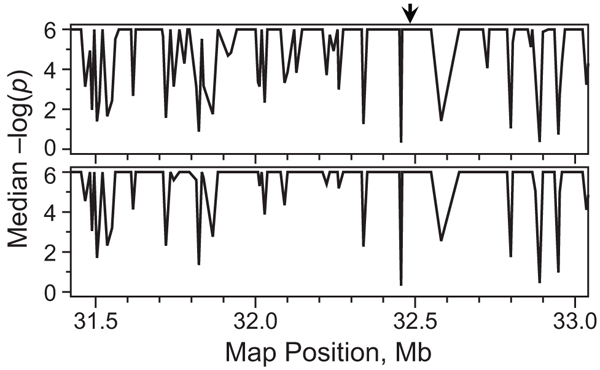
**Comparison of TDTEX and FBAT results nearHLA-DRB1**. Median negative log_10_(*p*) values from TDTEX (*top*) and FBAT (*bottom*) over 100 replicates are plotted for 150 consecutive markers near HLA-DRB1 (*arrow*).

As a preliminary step toward haplotype association analysis, we performed exploratory single-marker scans on case-control samples and trios. An initial scan with ASSOC on case-control samples gave evidence of an extremely strong genetic signal in the HLA region (data not shown). Evidence for association of most SNPs near DRB1 with RA was, overall, highly significant under both TDTEX and FBAT (Fig. 2). Nevertheless, a few SNPs interspersed among the markers in this region yielded *p*-values of unusually low significance by one or both methods. These results, which were consistent across replicates, could not be explained entirely by the informativity of the markers: the correlation between *p*-value and the number of families fully informative for the TDT (i.e., with both parents heterozygous) was low when the TDT had unusually low power. Specifically, of the eight markers with median *p*-values greater than 0.1, six were within the top three quintiles of the percentage of informative families (i.e., ≥36%). We expected a uniformly strong correlation between the available information on transmission and power of the TDT, and we were unable to explain this surprising result.

### Power analyses at HLA-DRB1 and locus D

Haplotype clustering substantially improved power to detect association with RA at the DRB1 locus when sex and the number of DRB1*04 alleles were included in the model as covariates. It was necessary to adjust for the strongest susceptibility allele to reduce the power to a level useful for comparing the haplotype- and cluster-based approaches. Sex was also included to reduce the contribution of locus C, which affects RA only in females. Despite these adjustments, association was detected with 70% power at *p *< 0.05 without clustering in samples of 150 each cases and controls (Table 1). Power was roughly equivalent with six- or eight-SNP haplotypes flanking DRB1, and was optimal at ε = 0.5 and *MinPts *= 0.25. Improvement in performance with clustering was considerable both at the 0.05 and 0.01 significance levels, with a greater than 20% increase at a nominal type I error of 0.01. Clustering reduced the average degrees of freedom of the score test by approximately 60%, providing ample opportunity for increasing power, provided that the haplotypes are grouped in a manner consistent with the evolution of disease-causing mutations.

**Table 1:** Power of haplotype- and cluster-based association analyses at the HLA-DRB1 locus^*a*^

	Power^*b*^		
		
	α = 0.05	α = 0.01	Mean d.f.^*c*^
*6 Markers*^*d*^			
Haplotypes	0.672	0.379	9.36
Clusters	0.812	0.593	3.65
*8 Markers*^*e*^			
Haplotypes	0.654	0.356	10.07
Clusters	0.822	0.597	3.94

^*a*^Sex and number of DRB1*04 alleles were included as covariates in all analyses.

^*b*^Proportion of 1000 data sets of 150 each cases and controls giving *p*-values below nominal α.

^*c*^Mean degrees of freedom from the score test for association.

^*d*^SNPs 3435–3440.

^*e*^SNPs 3433, 3435–3441.

In contrast, haplotype clustering only marginally improved the performance of the score test for haplotypes near locus D, some 5 cM from DRB1 (Table 2). Overall power was much reduced at this location, relative to DRB1: no adjustment for covariates was necessary, and a sample size of 500 cases and controls was required to obtain enough power to make comparisons. Here, extending the marker set from six to eight SNPs greatly enhanced our ability to detect association. Although maximal power was obtained at different values of ε than for the DRB1 analyses, performance of the cluster-based test was nearly as great at ε = 0.5. Whereas clustering reduced the mean d.f. of the score test by half when six markers were provided, it only condensed the parameters in the eight-SNP analysis by about 30%, which may have contributed to the diminished improvement of performance with clustering, relative to that observed at the DRB1 locus.

**Table 2:** Power of association analyses at Locus D^*a*^

	Power, no covariates		Power, adjusted^*b*^		
		
	α = 0.05	α = 0.01	α = 0.05	α = 0.01	Mean d.f.^*c*^
*6 Markers*^*d*^					
Haplotypes	0.240	0.088	0.059	0.009	9.04
Clusters, ε = 0.4^*e*^	0.272	0.104	--	--	4.48
Clusters, ε = 0.5	0.271	0.098	0.079	0.022	4.43
*8 Markers*^*f*^					
Haplotypes	0.448	0.206	0.112	0.025	14.37
Clusters, ε = 0.7^*e*^	0.470	0.239	--	--	10.26
Clusters, ε = 0.5	0.448	0.236	0.135	0.019	10.26

^*a*^Power was measured over 800 data sets of 500 each cases and controls.

^*b*^Sex and number of DRB1*04 alleles were included as covariates.

^*c*^See Table 1. Values shown are from analyses without covariates; mean d.f. from adjusted analyses were nearly identical.

^*d*^SNPs 3914–3919.

^*e*^Value of ε maximizing power for score test without covariates.

^*f*^SNPs 3913–3920.

Ability to detect association at locus D was markedly reduced with adjustment for sex and number of DRB1*04 alleles, with power at the 0.01 significance level falling almost to background (Table 2). This observation strongly suggests that DRB1 and locus C are providing most of the genetic signal at locus D. Low but significant LD (|D'| < 0.1; LOD score > 2 for H_0_: D' = 0) was observed between SNP 3437 at DRB1 and SNP 3917, 1.6 kb from locus D (data not shown). Given the overwhelming effect of DRB1 on RA, this small level of LD may explain the association between RA and haplotypes at locus D.

### Assessment of type I error

False-positive rates measured at HLA-DRB1 in the absence of covariates matched expectation when trait values were permuted relative to genotypes, both with and without haplotype clustering, as did those measured at locus D. With adjustment for sex and DRB1*04 alleles, cluster-based score tests again yielded proper type I error, whereas the test was somewhat conservative without clustering, returning a false-positive rate of about 0.025 at a significance level of 0.05. Results were similar for all six- and eight-marker haplotypes examined in power analyses (data not shown). Thus, our cluster-based score test appears to be robust to the severe dHWE encountered within the HLA region.

To further assess the validity of our approach, we performed score tests of association at four levels of haplotype diversity in regions distant from the HLA region (see Methods). Both the haplotype- and cluster-based approaches were valid at all diversity levels for sets of six and eight adjacent SNPs (Table 3). However, in the absence of clustering, as haplotype diversity increased the test became exceedingly conservative, with type I error rates of 1% or less at a nominal 0.05 significance level at the highest diversity level. Clustering greatly reduced this tendency, such that no clear downward trend in type I error occurred with six-SNP haplotypes, and only a modest decrease with eight-SNP haplotypes.

**Table 3:** Type I error of score tests, as a function of haplotype diversity

		Haplotype analyses			Cluster analyses		
			
Diversity	Haplotypes^*a*^	α = 0.05	α = 0.01	d.f.^*b*^	α = 0.05	α = 0.01	d.f.^*b*^
*6 Markers*							
Low	4.2	0.031	0.001	3.17	0.060	0.012	1.21
Medium	10	0.022	0.003	8.70	0.035	0.003	4.02
High	17	0.015	0.001	15.40	0.035	0.002	7.12
Very high	28	0.010	0.002	23.02	0.040	0.007	10.06
*8 Markers*							
Low	5.2	0.027	0.006	4.18	0.042	0.007	1.00
Medium	15	0.030	0.003	11.53	0.047	0.010	4.81
High	25	0.011	0.000	21.28	0.038	0.005	8.82
Very high	48	0.002	0.000	40.15	0.025	0.001	23.18

^*a*^Mean haplotype diversity over samples of 200 each RA cases and controls taken from 50 different Problem 3 replicates.

^*b*^Degrees of freedom from the score test averaged over 1000 samples of 150 each cases and controls analyzed with adjustment for sex and DRB1*04 alleles.

## Discussion

In summary, incorporation of haplotype clustering by the procedure of Li and Jiang [1] noticeably improves the power of the association mapping approach of Schaid et al. [3] to detect association with RA at the DRB1 locus (with adjustments to reduce signal strength), but only minimally improves power at locus D. In general, we expect clustering, in the presence of allelic heterogeneity, to improve performance of the score test and to enhance our ability to identify causative variants. Clustering also promises to increase power of the test in regions with extensive haplotype diversity by grouping rare haplotypes and thus reducing the degrees of freedom of the score test. However, because the Problem 3 data were not simulated under a coalescent model incorporating independent disease-causing mutations at DRB1, we could not directly test these hypotheses. Similarly, haplotype analysis would not necessarily be expected to improve upon single-SNP association methods given data simulated in this manner, especially at a trait locus as overwhelmingly influential as DRB1. Indeed, at least two other GAW15 studies did not obtain more significant results from haplotype analysis than with single-locus approaches [15,16].

Comparisons of the two approaches at these loci suggest guidelines for selecting operating parameters for HapMiner. Although performance was optimized at several values of ε, setting ε to 0.5 provided near-optimal results in all situations. The choice of *MinPts *affected performance very little at the relatively large range of ε displayed here. At small values of ε, however, *MinPts *may significantly affect the degree of clustering (data not shown). Limiting the extent of clustering by setting *p*_min _relatively small prevents "overclustering," in which haplotypes not recently diverged are grouped together, but also reduces the potential advantage of clustering. In practice, a reduction in power on clustering haplotypes is indicative of overclustering (data not shown; RPI, unpublished results). The Shannon information criterion employed by Tzeng et al. [13,17] for determining "core" haplotypes may also prove useful for limiting clustering of common haplotypes by the HapMiner algorithm. This method differs from that of Tzeng et al. [13] in that clustering is based on the distance metric rather than on an evolutionary model. In addition, less common haplotypes are not necessarily grouped with the most common ones, allowing widely diverged haplotypes to remain distinct.

Our work provides evidence that the GLM framework for association mapping is robust to severe departures from HWE under the null model. However, the GLM approach appears to be sensitive to adjustment with a covariate that is very tightly correlated with the trait, in that it may lose power in the presence of more extensive haplotype diversity, although clustering decreased this sensitivity, most likely by reducing the number of coefficients. It is possible that removing the multiplicative effect of HLA-DRB1*04 alleles on the odds of RA also extracted most of the trait information, perhaps changing the null distribution of the score statistic.

The apparent association at locus D appears to be largely due to HLA-DRB1 and locus C. The Problem 3 data appear to be unusual in that one locus exerts such a strong effect on the disease of interest that association is clearly discernible from a distance of over 5 cM. It is not surprising that our ability to detect locus D was marginal. The association study design is predicated on the "common disease-common variant" hypothesis [18], which posits that complex disease is characterized by small disease-locus effects for ancient, common alleles, and rampant locus heterogeneity. Locus D, on the other hand, has a very low disease allele frequency (0.008), and although the increase in risk is large with each disease allele, not enough susceptible genotypes were available in the case population to detect it.

## Competing interests

The author(s) declare that they have no competing interests.
